# HIV-1 Drug Resistance Mutations in Children: A Systematic Review of Global Genotypic Evidence (2014–2026)

**DOI:** 10.3390/pathogens15090958

**Published:** 2026-09-09

**Authors:** Ghita Jouf, Alae Bekkouri Alami, Najwa Maazaz, Soumia Benchekroun, Elmir Elharti, Imane Belbacha, Hassan Ihazmad, Rachid El Jaoudi, Rachid Benhida, Zineb Rchiad, Hicham Oumzil

**Affiliations:** 1 Medical Biotechnology Laboratory, Faculty of Medicine and Pharmacy, Mohammed V University in Rabat, Rabat 10100, Morocco; ghitajouf20@gmail.com (G.J.);; 2 Faculty of Medical Sciences (FMS), Mohammed VI Polytechnic University, Benguerir 43150, Morocco; 3 Chemical and Biochemical Sciences (CBS), Mohammed VI Polytechnic University, Benguerir 43150, Morocco; 4 Pediatric Infectious Disease Clinic, Ibn Sina University Hospital, Rabat 10100, Morocco; 5 Virology Department, Institut National d’Hygiène, Rabat 10100, Morocco; 6 CoreLab, Mohammed VI Polytechnic University, Benguerir 43150, Morocco

**Keywords:** HIV-1, drug resistance mutations, children, antiretroviral therapy, genotypic resistance, HIV-1 subtypes, systematic review

## Abstract

HIV-1 infection in children remains a major public health challenge, particularly in resource-limited settings where access to antiretroviral therapy (ART) and routine virological monitoring is limited. The emergence of HIV-1 drug resistance mutations (DRMs) compromises treatment efficacy and threatens long-term therapeutic outcomes. A systematic literature review was conducted using PubMed and Scopus to identify studies published from 2014 onward reporting HIV-1 genotypic resistance profiles in children receiving ART. Eligible studies included original research using Sanger sequencing or next-generation sequencing (NGS). Due to substantial methodological heterogeneity in study design, sequencing approaches, patient populations, and outcome reporting, a meta-analysis was not performed. The review therefore provides a narrative synthesis of resistance patterns and subtype distribution. Included studies involved children aged 2.1–16 years and sample sizes ranging from 3 to 1080 participants. Resistance mutations were most frequently observed in the NNRTI and NRTI classes, particularly K103N and M184V, whereas protease inhibitor and integrase inhibitor resistance remained less common. HIV-1 subtype distribution showed marked geographic variation, with CRF01_AE predominating in Asia, subtype C in Southern Africa, CRF02_AG and CRF06_cpx in West Africa, and subtype B in Europe and the Americas. These findings highlight the substantial burden of pediatric HIV-1 drug resistance and the need for strengthened surveillance and optimized region-specific treatment strategies.

## 1. Introduction

HIV-1 infection in children remains a critical global public health challenge. According to UNAIDS, approximately 1.4 million children under 15 years of age were living with HIV in 2023, with more than 90% residing in sub-Saharan Africa. Each year, an estimated 120,000 new pediatric infections occur, the vast majority resulting from mother-to-child transmission (MTCT) during pregnancy, delivery, or breastfeeding. Although programs for the Prevention of Mother-to-Child Transmission (PMTCT) primarily rely on maternal and infant antiretroviral (ARV) prophylaxis and have substantially reduced vertical transmission rates, millions of children nonetheless live with chronic HIV-1 infection and require lifelong treatment.

The widespread scale-up of antiretroviral therapy (ART) over the past two decades has greatly impacted the prognosis of children with HIV, reducing AIDS-related mortality and morbidity. Current pediatric regimens are based on three main drug classes—nucleoside/nucleotide reverse transcriptase inhibitors (NRTIs), non-nucleoside reverse transcriptase inhibitors (NNRTIs), and protease inhibitors (PIs)—with integrase strand transfer inhibitors (INSTIs) increasingly recommended in updated guidelines. However, the emergence and accumulation of drug resistance mutations (DRMs) represent a major obstacle to ART success, particularly in resource-limited settings where virological monitoring remains inconsistent and second-line options are scarce.

Compared to adults, children are facing unique virological vulnerabilities. The combination of an immature immune response, higher baseline viremia, and developmental limitations in hepatic and renal drug metabolism contributes to distinct pharmacokinetic characteristics in pediatric populations. These factors complicate the achievement of optimal drug concentrations and may result in subtherapeutic exposure, thereby promoting the selection of a resistant viral variant.

Additionally, children perinatally exposed to ARVs through PMTCT programs may harbor pre-existing resistance mutations at the time of treatment initiation, most notably NNRTI-associated mutations such as K103N and Y181C, which directly compromise the efficacy of first-line regimens. The specificity of this clinical and biological status underscores the urgent need for pediatric-specific surveillance of HIV drug resistance mutations (DRMs).

Despite their importance, data on DRMs among the pediatric population living with HIV remain fragmented, geographically heterogeneous, and underrepresented in the scientific literature relative to adult populations. Genotypic resistance testing, the gold standard for identifying DRMs through sequencing of the HIV-1 reverse transcriptase, protease, and integrase genes, is often not available or inconsistently applied in the most affected settings, leading to the establishment of critical blind spots in the global understanding of DRM epidemiology among children.

This narrative systematic review aims to address these gaps by synthesizing the available evidence on HIV drug resistance mutations among children living with HIV-1, with a particular focus on the prevalence, distribution, and patterns of reported DRMs.

Furthermore, we underscore persistent deficiencies in pediatric virological monitoring and advocate for the regional tailoring of therapeutic recommendations based on local HIV drug resistance profiles. The evidence generated by this study may contribute to the optimization of pediatric HIV treatment policies and aid in pleading for a system integration of DRM surveillance activities.

## 2. Materials and Methods

### 2.1. Data Source and Searches

Publications were obtained from PubMed/MEDLINE and Scopus/ScienceDirect using the following keywords: “HIV genotyping,” “antiretroviral therapy,” “resistance mutation,” “HIV-1”, and “children”. Keywords were combined using the Boolean operators “OR” and “AND.” Study inclusion criteria include randomized and non-randomized controlled trials, cohort studies, and cross-sectional studies published in French or English since 2014.

However, case reports, reviews, systematic reviews, meta-analyses, and editorials were excluded.

Data were presented according to sample type, geographic location (country/continent), ethnicity/race, age (infants vs. children), HIV-1 subtypes, ART exposure (naïve vs. treated), and HIV drug resistance mutations.

### 2.2. Study Selection

The study selection process was conducted in two phases. The first phase involved reviewing the titles and abstracts of the available articles. Two reviewers independently performed the initial screening to assess the potential relevance of each study according to the predefined inclusion criteria.

Following this initial step, articles deemed potentially relevant underwent further review. The full texts of the selected articles were independently assessed by the same two reviewers to confirm that each study adhered to the specific inclusion criteria defined for our research. Any disagreements were resolved through discussion and consensus.

The final list of included studies was reviewed and validated by the supervising author to ensure methodological rigor and consistency.

This two-step method ensured a systematic study selection process, combining an initial screening based on titles and abstracts with a thorough analysis of full-text articles, thereby maintaining a high standard of quality and relevance for the body of literature included in this review.

This study was designed as a systematic review without meta-analysis. The decision to forgo quantitative data pooling was based on the substantial heterogeneity identified across included studies, encompassing differences in study design (cross-sectional surveys, retrospective and prospective cohorts, and trial-derived observational studies), target populations (treatment-naïve children with pretreatment drug resistance (PDR) versus treatment-experienced children with acquired drug resistance (ADR), or mixed populations without stratification), sequencing methodologies (Sanger sequencing versus next-generation sequencing (NGS)), geographic and epidemiological contexts, and the format of resistance data reporting (proportions of individual mutations versus aggregate resistance profiles). In this context, statistical pooling would have introduced spurious precision and obscured clinically meaningful regional and population-level variation. A narrative synthesis was therefore considered the most appropriate and scientifically rigorous approach for characterizing the global landscape of HIV-1 drug resistance mutations in pediatric populations.

### 2.3. Risk-of-Bias Assessment

The methodological quality of included studies was assessed using an adapted risk-of-bias framework derived from established approaches for observational studies, including the Newcastle–Ottawa Scale and the Joanna Briggs Institute Critical Appraisal Tools. Three core domains were evaluated: selection bias, measurement bias, and confounding.

Selection bias was defined as the extent to which the study population reflects the target population of pediatric HIV drug resistance, including both pretreatment resistance (PDR) related to vertical transmission and acquired resistance (ADR) associated with antiretroviral treatment failure. Studies restricted to virological failure populations were not systematically considered at high risk, given their alignment with ADR-focused objectives, but were evaluated in terms of representativeness and generalizability.

Measurement bias refers to the accuracy and reliability of genotypic resistance detection. As the analysis focused on clinically relevant major drug resistance mutations, both Sanger sequencing and next-generation sequencing (NGS) were considered appropriate and comparable methodologies, with limited expected impact on the detection of major resistance patterns.

Confounding was assessed based on the potential influence of clinical and treatment-related factors, including ART exposure, duration of treatment, and Prevention Of Mother-To-Child Transmission (PMTCT). Given the descriptive objective of the study, which aimed to characterize resistance patterns broadly rather than infer causal relationships, confounding was considered to have a limited impact on the primary outcomes.

An overall risk of bias was assigned to each study based on the most critical domain, with particular emphasis on sample size, representativeness, and analytical clarity.

### 2.4. Ethics

This study does not require ethics approval, as it focuses solely on the review of previously published data. The systematic review is conducted on data extracted from public sources such as PubMed/MEDLINE and Scopus/ScienceDirect. These data have already undergone ethical review procedures upon their initial disclosure. By using this approach, the study complies with current ethical standards while maximizing the use of existing resources without involving new interactions with participants. This methodology promotes scientific transparency by consolidating and interpreting previously validated results.

## 3. Results

We followed PRISMA guidelines to conduct this systematic review. The review protocol was registered in the Prospective Register of Systematic Reviews (PROSPERO) under registration number CRD42024512303. For further details, the full PRISMA checklist can be consulted (Table S3).

**Figure 1:**
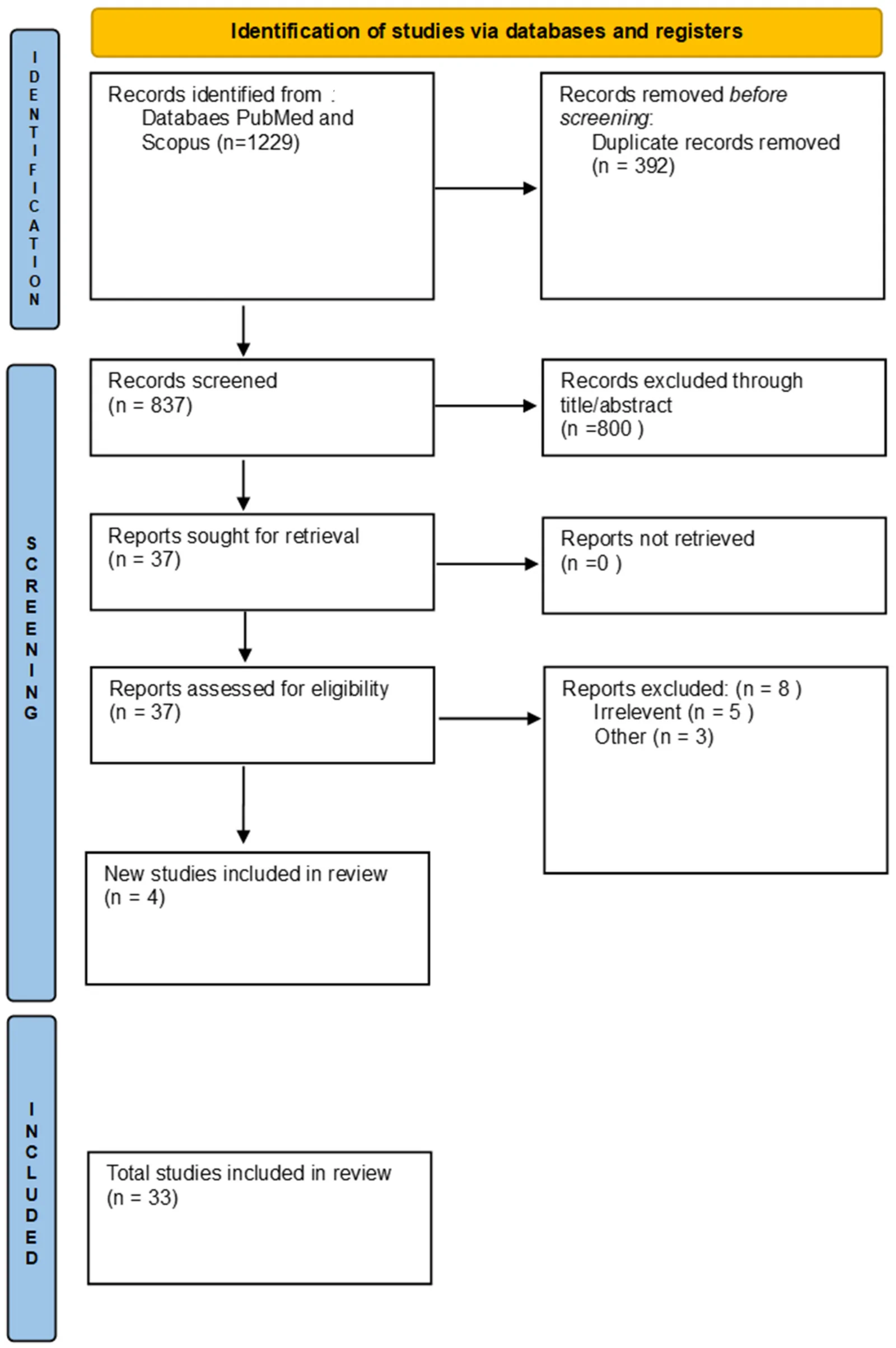
PRISMA 2020 flow diagram for new systematic reviews, which included searches of databases and registers only.

A total of 1229 records were identified from the PubMed and Scopus databases. Of these, 392 duplicate records were removed prior to screening. After screening, 837 records were reviewed, of which 800 were excluded based on the title or abstract. A total of 37 full-text articles were assessed for eligibility. Of these, 8 articles were excluded, including 5 due to irrelevance and 3 for other reasons, leaving 29 eligible studies. Four additional studies identified during the updated search process met the inclusion criteria and were incorporated into the review. Ultimately, a total of 33 studies were included in the systematic review (Figure 1).

The selection of studies for our systematic review provides a diverse overview of HIV data, covering a range of geographical contexts. Among the studies selected, we observe a global representation, with research conducted in countries in Africa (Kenya ([1,2,3]), Cameroon [4], Ethiopia [5,6], Southern Tanzania [7,8], Nigeria [9], Equatorial Guinea [10], Zambia [11], Democratic Republic of Congo [12], Togo [13,14], Uganda [15], Mali [16], Zimbabwe [17], sub-Saharan Africa [18], Ghana [19], Benin [20] and South Africa [21]), Asia (China [22] and India [23]), America (Uruguay [24], Brazil [25,26], Panama [27], Amazonas [28], United States [29], and Rhode Island [30]), Europe (France [31] and Russia [32]), and even one study encompassing North America, Europe and South Africa [33].

### 3.1. Characteristics of Included Studies

**Figure 2:**
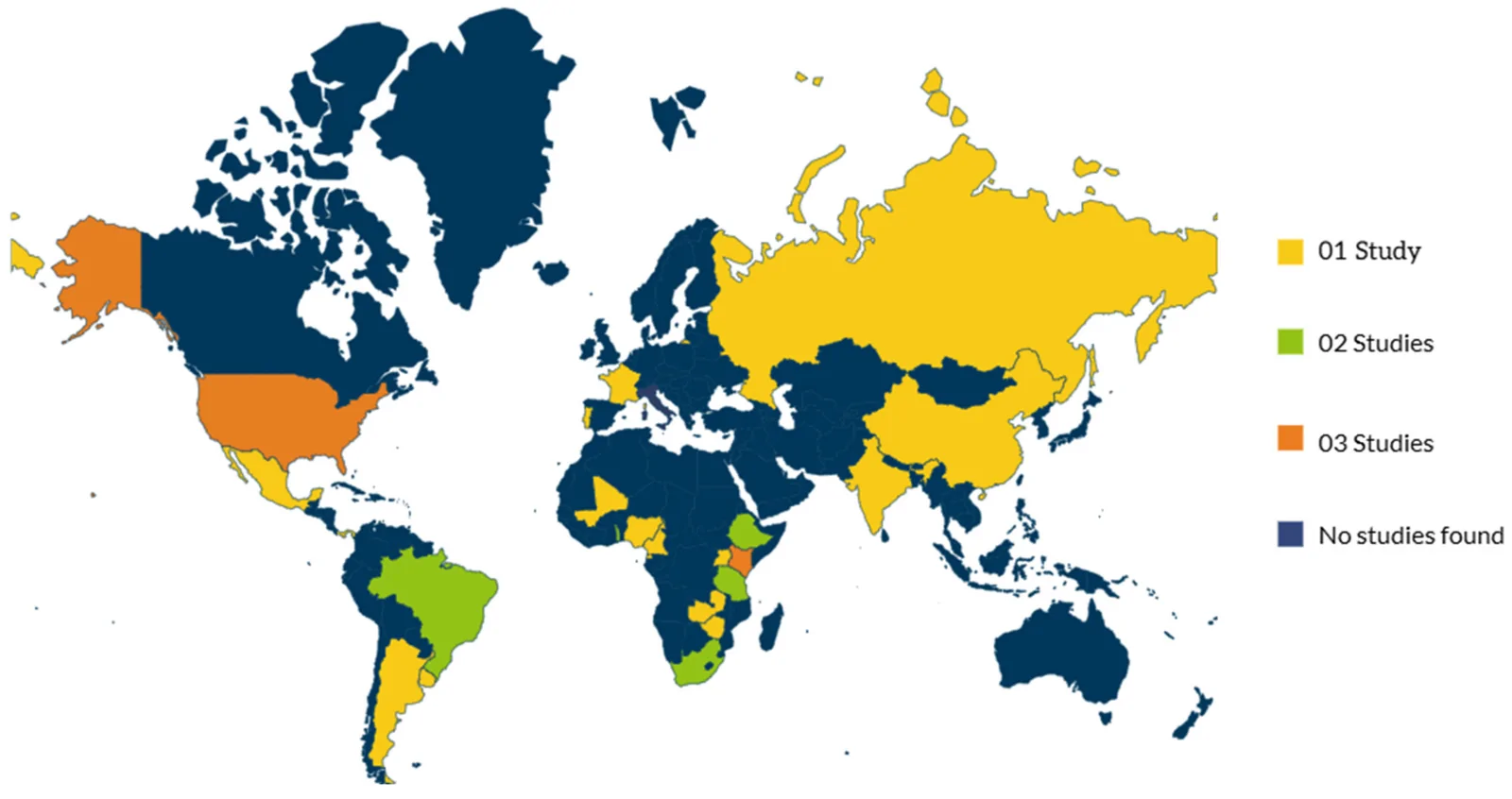
Geographical distribution of studies selected for the systematic review on antiretroviral resistance in pediatric patients.

The data in this systematic review covers regions from Africa, Asia, Europe, the Americas, and Oceania, with median ages ranging from 2.1 to 16 years and sample sizes varying widely (from 3 to 1080). The types of samples analyzed primarily include whole blood, plasma, and dried blood spots, with a majority using next-generation sequencing (NGS), although Sanger sequencing is also used in several cases (Table S1). The antiretroviral classes studied frequently include NRTIs, NNRTIs, PIs, and sometimes integrase inhibitors (INs). The diversity of viral subtypes identified is notable, with a predominance of circulating recombinant forms (CRFs) and subtypes such as A, B, C, D, G, or their recombinant forms. These data highlight the geographic, virological, and methodological heterogeneity in pediatric HIV resistance studies (Figure 2).

The analyzed studies show the distribution of resistance mutations to non-nucleoside reverse transcriptase inhibitors, nucleoside reverse transcriptase inhibitors, protease inhibitors, and integrase inhibitors. This data is derived from distinct studies conducted across multiple geographic regions.

**Figure 3:**
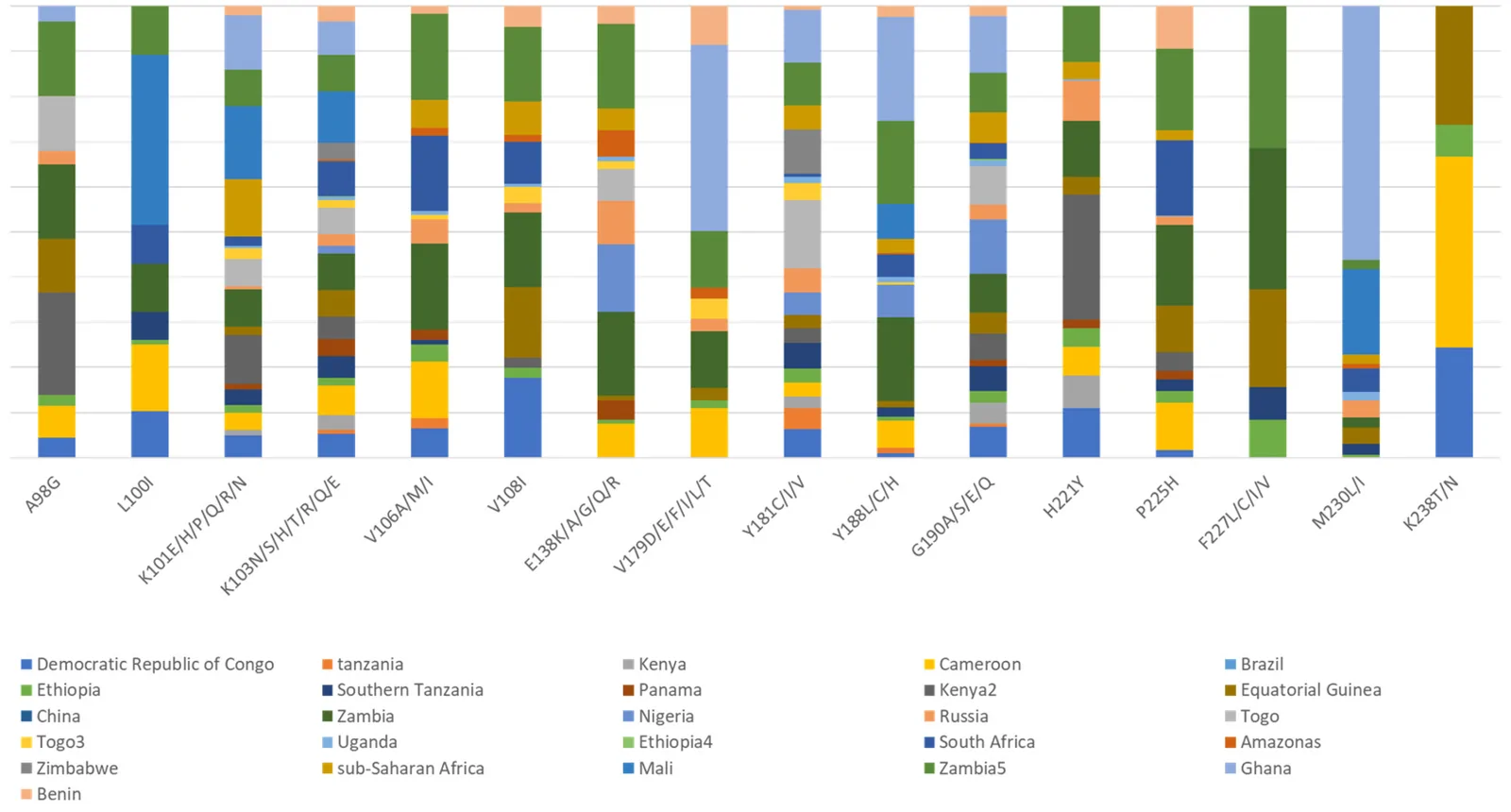
Prevalence of NNRTI-associated drug resistance mutations in HIV-1 sequence.

The distribution of the 28 major NNRTI resistance mutations varied markedly across regions and countries (Figure 3). In Africa, mutations at codon 103, including K103N/S/H/T/R/Q/E, were among the most frequently reported and predominated in several countries. Their highest prevalence was observed in Mali (90.90%), followed by Zambia (65.50%), South Africa (63.00%), Ghana (60.00%), Cameroon (52.30%), Equatorial Guinea (47.10%), Togo (47.22%), Kenya (40.47%), Benin (28.10%), and Tanzania (6.52%). However, the second most frequently detected mutation differed by country. Mutations Y181C/I/V were highly prevalent in Togo (58.33%), K101E/H/P/Q/R/N were reported in Kenya (40.47%), and V106A/M/I reached 31.50% in South Africa. In Tanzania, Y181C was the predominant NNRTI resistance mutation (17.39%), followed by V106I (4.34%), whereas G190A and Y188L were each detected at 2.17%. Mutations at codon 190, including G190A/S/E/Q, were also commonly reported in Nigeria (42.80%), Zambia (31.00%), Togo (30.55%), the Democratic Republic of the Congo (24.50%), and Southern Tanzania (20.00%). By contrast, Y188F, P225H, F227L/C/I/V, M230L/I, L234I, K238T/N, and M232H were generally uncommon, with reported prevalences below 5% in most settings. Ethiopia showed a more heterogeneous pattern, with Y181C/I/V detected at 11.80%, G190A/S/E/Q at 9.00%, V106A/M/I at 7.20%, and several other mutations at frequencies below 5%.

In Asia, the available data from China showed that K103N/S/H/T/R/Q/E was the most frequently reported NNRTI resistance mutation, with a prevalence of 26.52%, followed by Y181C/I/V at 14.39%. Other mutations, including V106A/M/I, Y188L/C/H, and P225H, were reported at prevalences of 10% or less, while several additional NNRTI resistance mutations were not detected.

In the Americas, three studies reported on NNRTI primary mutations. In Brazil, K103N/S/H/T/R/Q/E was the most prevalent 33.33%, followed by P225H 11.11%, with no other mutations detected. In Panama, K103N/S/H/T/R/Q/E also led at 10%, followed by E138K/A/G/Q/R at 6.70%, while several others including K101E/H/P/Q/R/N, V106A/M/I, G190A/S/E/Q, H221Y, and P225H appeared at frequencies below 5%, and the rest were absent. In the Amazonas, eight mutations were detected, all under 10%, with E138K/A/G/Q/R being the highest (9.40%), followed by moderate levels of V90I, K103N/S/H/T/R/Q/E, V106A/M/I, and V179D/E/F/I/L/T. Less common mutations such as Y188L/C/H, G190A/S/E/Q, and M230L/I showed a prevalence of 0.85%, while others were not detected.

In the European context, Russia was the only country with relevant data. All prevalences were at 20% or below, with K103N/S/H/T/R/Q/E and Y181C/I/V tied for the highest prevalence at 20%. This was followed by E138K/A/G/Q/R at 15%, G190A/S/E/Q at 12%, and A98G, K101E/H/P/Q/R/N, V108I, V179D/E/F/I/L/T, H221Y, P225H, and M230L/I, all with prevalences of 10% or below, while the remaining mutations had a prevalence of 0%.

Across the studies reviewed, the K103N/S/H/T/R/Q/E mutation is among the most frequently reported NNRTI primary mutations and is particularly dominant in Africa, China, and the Americas. Other mutations, such as Y181C/I/V and G190A/S/E/Q, appear at lower frequencies, with varying patterns observed in each region.

**Figure 4:**
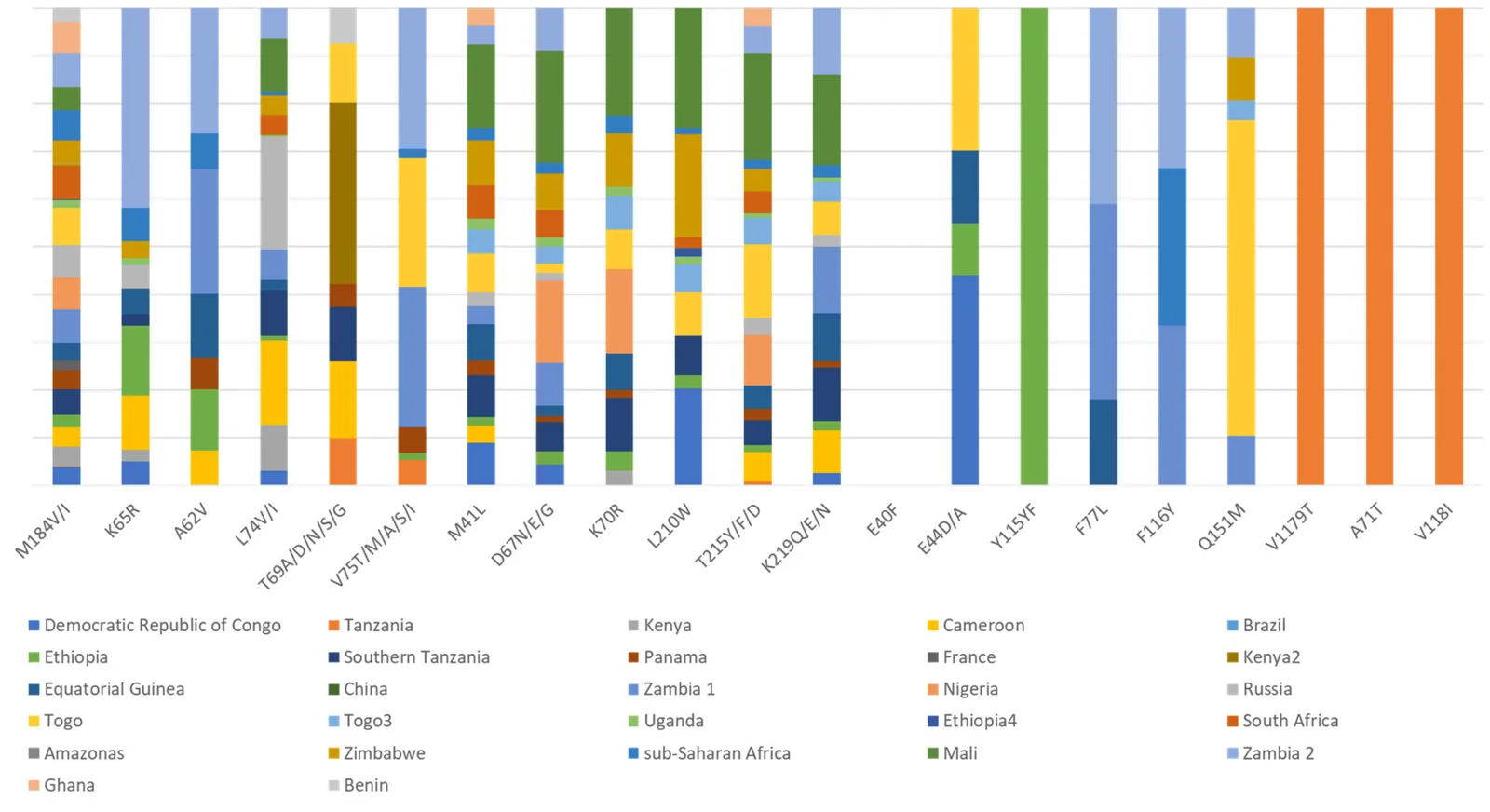
Prevalence of NRTI-associated drug resistance mutations in HIV-1 sequence.

The second set of mutations studied included 42 NRTI primary mutations, which also showed varied prevalences and patterns (Figure 4).

Across all African countries studied, M184V/I was consistently the most prevalent NRTI primary mutation, appearing as the top mutation in almost every country. Its prevalence ranged from 18.92% in Uganda to 88.88% in Togo and 75% in Ghana, making it by far the most dominant mutation throughout the region. The second most common mutations varied between countries. For example, T215Y/F/D took the second spot in Mali (60.60%) and Togo (41.66%). In Zambia, K65KR was the second most prevalent mutation, with a frequency of 34.50%. D67N/E/G came in second in Nigeria (33.30%), while K70E/G/Q/T/N/S held the second position in the Democratic Republic of Congo (14.30%) and Zambia (32.80%). In Ethiopia, due to a broad diversity of mutations, multiple mutations followed at lower frequencies after M184V/I (30.10%).

Across the dataset, several mutations consistently showed very low prevalences (≤5%). These included F77L, Q151M, E204AK, F116Y, K223R/E, L109I, L234I, T139K, and D218E, which appeared mostly in Zambia, typically below 5%. Mutations such as K65R, L74V/I, K219Q/E/N, T69D/N/S/G, M41L, D67N/E/G, L210W, and K70R appeared in multiple countries but remained at low frequencies in some, often under 5% in Uganda, Zimbabwe, and Equatorial Guinea.

Some mutations were either unique to a single country or appeared in only two countries. M184MV was seen in Togo and Ethiopia. K219KQ appeared only in Ethiopia and South Africa. K70KR and Y115YF were found exclusively in Ethiopia. Additionally, T139K, L109I, L234I, D218E, K223E, K223R, and E204AK were detected solely in Zambia.

Moving to Asia, only one country reported relevant prevalences of NRTI primary mutations, which is China. A total of five mutations were detected, with M184V/I taking the lead at a prevalence of 30.05%. The remaining mutations showed low prevalences: L74V/I, S68G/N/D, and D67N/E/G were all detected at 3.79%, while Y115F was found at 3.28%. The other mutations were not detected.

Turning to the Americas, only three studies provided data on NRTI primary mutations. In Brazil, three mutations were detected. The most relevant mutation was T215Y/F/D, with a prevalence of 44.44%, followed by M184V/I at 33.33%, and M41L at 22.22%, while the rest were at 0%. In Panama, a higher number of mutations was detected. The highest prevalence was for M184V/I at 48.90%, while the remaining detected mutations had low prevalences: 6.70% for T215Y/F/D, 4.40% for M41L, and 2.20% for A62V, T69/N/S/G, V75T/M/A/S/I, D67N/E/G, K70G, and K219Q/E/N. In the Amazonas, only one detected mutation was reported: M184V/I, with a prevalence of 1.71%.

As for Europe, a lack of data was again observed, with only the Russian study being relevant. It reported the presence of nine mutations. The highest prevalence was for M184V/I at 80%, followed by L74V/I at 32%, and Y155F at 20%. The remaining detected mutations were M184V/I, K70E/G/Q/T/N/S, M41L, D67N/E/G, T215Y/F/D, and K219Q/E/N, with prevalences falling at 10% or below, while the French study solely reported the presence of M184V/I with a prevalence of 21.70%.

**Figure 5:**
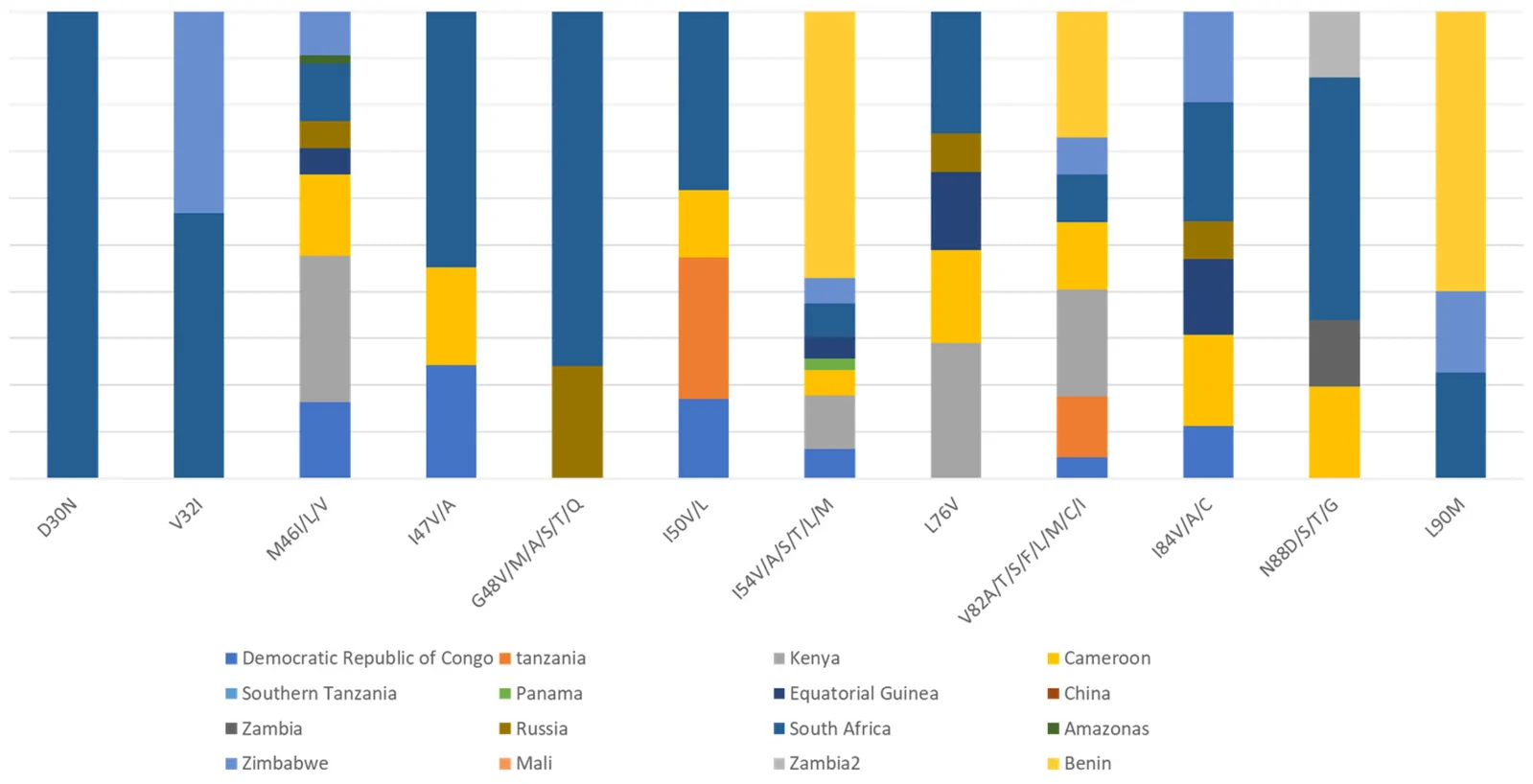
Prevalence of PI-associated drug resistance mutations in HIV-1 sequence.

Across the studies reviewed, NRTI primary mutations showed varying prevalences, with M184V/I consistently being the most prevalent mutation in all studied continents (Figure 5). Other mutations, such as T215Y/F/D and K65KR, also appeared frequently, though their ranking varied by country, while K70KR and Y115YF were found exclusively in Ethiopia.

The third set of mutations studied included 28 PI primary mutations, which also showed diverse patterns and prevalences of mutations (Figure 5).

Across African countries, M46I/L/V was the most consistently prevalent PI primary mutation. It appeared in the DRC (8.30%), Kenya (16%), Cameroon (8.90%), Equatorial Guinea (2.80%), and Zimbabwe (4.80%). Another frequent mutation was I54V/A/S/T/L/M, found in the DRC (5.60%), Kenya (10%), Cameroon (4.80%), Equatorial Guinea (4%), and Zimbabwe (4.80%). V82A/T/S/F/L/M/C/I was also among the more common mutations, recorded in Kenya (14%), Cameroon (8.90%), and Zimbabwe (4.80%). I84V/A/C appeared in the DRC (2.80%), Cameroon (4.80%), and Equatorial Guinea (4%).

Less frequently detected mutations included I47V/A, I50V/L, N88D/S/T/G, Q58E, L24I/F/M, L19F/I/V/R/Y, K20R/I/M/T/V, L10F/I/V/R/Y, V32I, A71V/T/I/L, and M36I, generally observed at low prevalences between 1.72% and 5.60%, and often restricted to one or two countries.

Some mutations were found uniquely in one or two countries. A71V/T/I/L (60.60%) and M36I (42.42%) were exclusive to Mali. Q58E (5.17%) was detected only in Zambia, along with L19F/I/V/R/Y (1.72%) and K20R/I/M/T/V (1.72%). L10F/I/V/R/Y (4.80%), V32I (4.80%), 184V/A/C (4.80%), and L90M (4.80%) appeared solely in Zimbabwe.

Overall, the prevalences of PI mutations were generally low, and out of the 28 possible mutations studied, only a limited number were detected in each country, with most mutations appearing in just one or two locations. Cameroon and Zimbabwe showed the highest diversity, with eight and seven mutations, respectively, while Mali and Zambia reported far fewer mutations despite high percentages in Mali and Benin. It is important to highlight that several countries such as Ethiopia, Nigeria, Southern Tanzania, Togo, and Uganda had no available data on PI primary mutations, leaving important gaps in the regional resistance landscape.

The Asian continent did not show high prevalences either. In China, only three mutations were recorded, with the highest prevalence being 0.51% for both M46I/L/V and V82A/T/S/F/L/M/C/I, while the remaining mutation had a prevalence of 0.25%.

In the Americas, data on PI primary mutations was extremely limited, with only two mutations recorded. I54V/A/S/T/L/M was detected in Panama at a low prevalence of 2.20%, while M46I/L/V appeared in Amazonas with an even lower prevalence of 0.85%. No other mutations were reported in these two countries, and no data was available for the rest of the region.

Similarly, in Europe, data was extremely limited, with only four mutations reported in Russia, all at very low prevalences. The highest was M46I/L/V at just 3%, followed by G48V/M/A/S/T/Q, L76V, and I84V/A/C, each recorded at 2%.

**Figure 6:**
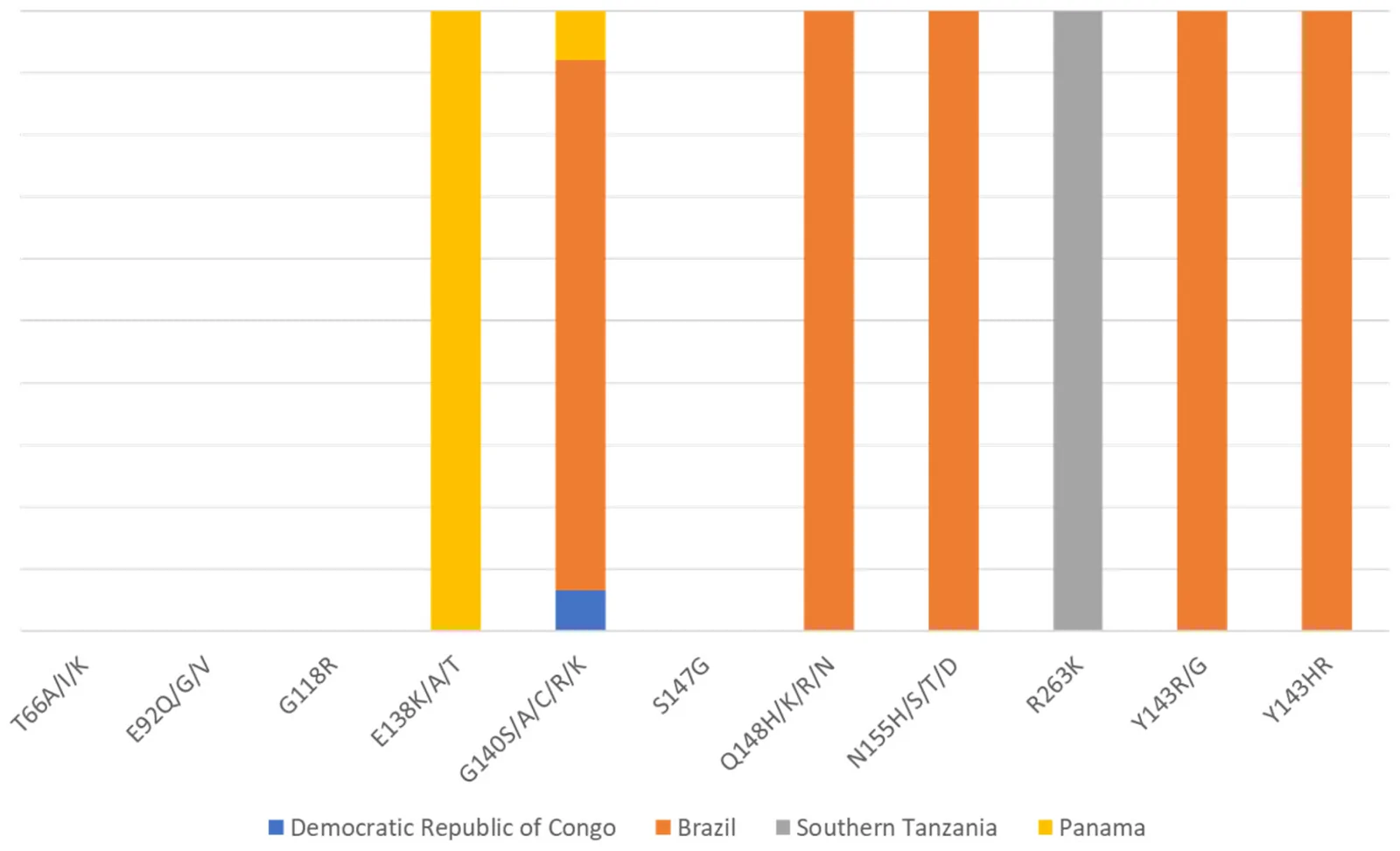
Prevalence of INSTI-associated drug resistance mutations in HIV-1 sequence.

PI primary mutations showed low prevalences and limited diversity globally, with Africa reporting the highest numbers, notably for M46I/L/V and I54V/A/S/T/L/M, while Mali stood out for exceptionally high rates (Figure 6). Asia, the Americas, and Europe had sparse data, with only a few low-prevalence mutations recorded, leaving significant gaps in many regions.

Regarding INSTI mutations, where 26 mutations were studied, the patterns of prevalence and distribution revealed similarly limited data and low mutation frequencies across most regions (Figure 6). Once again, starting with Africa, overall mutation prevalences were low, and only two countries had reported data: Equatorial Guinea showed the highest prevalence with T97A at 7.40%, while in the DRC, it appeared at 2%, alongside Q95K and G140S/A/C/R/K at 2.50%, and E157Q at 1.90% in Equatorial Guinea. Notably, no data was reported in the remaining studied countries.

In Asia, China was the only country with available data, where the most notable mutation was E157Q with a prevalence of 17%, while the remaining 13 mutations including H51Y, E92Q/G/V, Q95K, T97A, G118R, E138K/A/T, G140S/A/C/R/K, S147G, Q148H/K/R/N, N155H/S/T/D, G163R/K, S230R, and R263K each showed prevalences below 1%.

A similar situation was observed in the Americas, where data on integrase inhibitor resistance were available from Panama and Brazil. In Panama, all detected mutations showed relatively low prevalences, with E157Q being the most frequent (4.50%), followed by T97A, G140S/A/C/R/K, and Y143C/R/H/K/S/G/A (3.00%), while E138K/A/T and Q148H were each detected at 1.50%. In Brazil, a separate study reported substantially higher frequencies of major INSTI resistance mutations, with Q148H (35.48%) and G140S (32.25%) being the most prevalent, followed by N155H (22.58%) and Y143R/G (9.77%).

Europe showed a similar pattern, with France as the only country providing data. Just three mutations were recorded, with L74M/I/F having the highest prevalence at 9.70%, followed by E157Q at 6.50% and T97A at 1.60%.

Across the continents, integrase inhibitor mutations showed limited data availability and generally low prevalences. E157Q was the most recurrent mutation, detected in all regions with varying frequencies, while T97A and G140S/A/C/R/K appeared in multiple continents. Most other mutations were either rare or specific to single countries with large data gaps.

### 3.2. Subtype Distribution Analysis

The summary of global HIV subtype distribution data reveals marked diversity across several regions. In Africa, the reported subtypes primarily include A, C, D, G, F2, H, and K, as well as many recombinant forms such as CRF02_AG, CRF06_cpx, CRF13_cpx, CRF11_cpx, CRF22_01A1, CRF26_AU, CRF37_cpx, CRF10_CD, and various URFs. In West and Central Africa (Cameroon, Nigeria, Mali, Equatorial Guinea, and Togo), there is a great diversity with a predominance of complex recombinants. In East Africa (Kenya, Uganda, and Ethiopia), subtypes A1, C, D, and their recombinants are the most common. In Southern Africa (Zambia, Zimbabwe, Tanzania, and South Africa), subtype C dominates and is sometimes associated with recombinants like CRF10_CD.

### 3.3. Risk-of-Bias Synthesis

The overall methodological quality of the included studies was heterogeneous but generally acceptable for descriptive synthesis. Selection bias was predominantly classified as low to moderate, reflecting the alignment of most studies with the predefined target population of pediatric HIV drug resistance, encompassing both PDR and ADR contexts. Higher selection bias was observed in studies with very small sample sizes or limited representativeness, particularly single-center or retrospective cohorts.

Measurement bias was consistently low across studies, as genotypic resistance testing methods were deemed robust for the detection of major drug resistance mutations. Differences between Sanger sequencing and NGS were not expected to significantly influence the identification of clinically relevant resistance profiles.

Confounding was generally classified as moderate. Although several studies included heterogeneous populations in terms of treatment exposure and clinical history, this heterogeneity was considered inherent to pediatric HIV resistance epidemiology and did not substantially compromise the descriptive assessment of resistance patterns.

Overall, the risk of bias was primarily driven by study design characteristics, particularly sample size and representativeness, rather than laboratory or methodological limitations (Table S2).

## 4. Discussion

We reviewed studies containing substantial information on antiretroviral drug resistance (HIVDR) in pediatric populations. These works were selected for their remarkable contribution to our understanding of this critical public health issue. Most investigations originated from sub-Saharan Africa (SSA), reflecting the region’s high pediatric HIV burden as well as persistent challenges in access to health services and antiretroviral therapy. Recent meta-analytical data confirm this regional disparity, showing that the pooled prevalence of pretreatment drug resistance (PDR) in SSA is significantly higher at 39.13% compared to 22.56% in other regions [34]. Countries such as Kenya, Cameroon, Ethiopia, Southern Tanzania, Nigeria, Equatorial Guinea, Zambia, and the Democratic Republic of Congo each offer distinct perspectives shaped by their epidemiological contexts.

Beyond Africa, additional insights arose from studies conducted in Asia, in particular in China, as well as from the Americas, including Uruguay, Brazil, and Panama. European data, although limited, were represented by studies from France and Russia. All of these geographically diverse studies provide a global view of pediatric HIV drug resistance, which currently shows a global pooled prevalence of 31.94% for PDR and an alarming 61.43% for acquired drug resistance (ADR) among children experiencing virological failure [34].

Understanding the genetic diversity of HIV across regions remains essential for improving global HIV management strategies. Consistent with previous observations [35,36], this review highlights considerable variability in subtype distribution and treatment resistance profiles. Across the studies reviewed, a broad spectrum of mutations was documented, each illustrating different patterns of drug selection pressure. The meta-analysis underscores that resistance is primarily driven by non-nucleoside reverse transcriptase inhibitor (NNRTI) mutations, which account for 28.38% of PDR and 65.17% of ADR cases globally [34].

Although the present review was not designed to investigate the relationship between HIV-1 subtype diversity and the selection of specific drug resistance mutations, subtype-related genetic variability may partly explain the regional differences observed in resistance profiles. Previous studies have suggested that certain HIV-1 subtypes and circulating recombinant forms (CRFs) differ in their evolutionary pathways under antiretroviral drug pressure, potentially influencing the emergence and persistence of particular resistance mutations [37]. However, the considerable heterogeneity of the included studies, together with differences in treatment regimens, sequencing methodologies, and study populations, precluded a robust assessment of subtype-specific resistance patterns. Further studies specifically designed to evaluate the interaction between HIV-1 genetic diversity and resistance evolution in pediatric populations are therefore warranted.

Among NNRTIs, mutations such as K103N/S/H/T/R/Q/E and G190A/S/E/Q were particularly prevalent. The high occurrence of K103N in sub-Saharan Africa likely reflects strong selective pressure resulting from the extensive use of nevirapine (NVP) and efavirenz (EFV) in first-line pediatric regimens. Crucially, the history of Prevention of Mother-to-Child Transmission (PMTCT) exposure plays a decisive role: children exposed to PMTCT prophylaxis exhibit a PDR rate of 43.23%, nearly triple the 19.40% observed in those without such exposure. Y181C/I/V and G190A/S/E/Q also appeared frequently, indicating prolonged exposure to NNRTIs in these regions. The persistence of these mutations, such as Y181C and K103N, may be further explained by the high viral fitness of the variants harboring them [34].

Conversely, rare mutations such as F227L/C/I/N or K238T/N require the accumulation of several sequential changes or are associated with second-generation NNRTIs such as etravirine (ETR) or rilpivirine (RPV)—which remain less accessible in resource-limited settings. Their scarcity may also relate to reduced viral fitness, limiting persistence in the absence of direct drug pressure. Finally, while the rollout of dolutegravir (DTG) has improved outcomes, the emergence of resistance to integrase strand transfer inhibitors (INSTIs), rising from 0% in 2015 to 5.5% in 2023 for ADR, necessitates the urgent implementation of genotypic resistance surveillance to safeguard future pediatric treatment strategies [34].

Regarding NRTIs, M184V/I, K65R, D67N/E/G, T215Y/F/D, and M41L were among the most common mutations across countries. Their prevalence reflects long-standing use of lamivudine (3TC), stavudine (d4T), and zidovudine (AZT). M184V/I remains especially dominant due to its rapid selection under 3TC/FTC pressure, despite its known fitness cost, which can, paradoxically, be clinically advantageous when combined with other ARVs.

The frequent reporting of M184V and K103N among the included studies has important clinical implications for pediatric HIV management. M184V is associated with high-level resistance to lamivudine (3TC) and emtricitabine (FTC), two nucleoside reverse transcriptase inhibitors that have been widely used in first-line pediatric antiretroviral therapy (ART) regimens [38]. Similarly, K103N confers high-level resistance to the first-generation non-nucleoside reverse transcriptase inhibitors (NNRTIs), particularly efavirenz (EFV) and nevirapine (NVP), potentially compromising the effectiveness of these regimens [39]. The frequent detection of these mutations highlights the importance of timely virological monitoring and genotypic resistance testing, whenever available, to guide treatment optimization [39]. In resource-limited settings, where routine resistance testing remains limited, continuous HIV drug resistance surveillance is essential to support evidence-based treatment guidelines and facilitate the implementation of antiretroviral regimens with a higher genetic barrier to resistance, such as dolutegravir-based therapies [18].

Rare mutations such as E40F were not detected in the included studies, consistent with their high genetic barrier and association with multi-nucleoside resistance patterns rarely selected under current regimens (notably in combination with Q151M).

Mutations like Y115YF, observed only in the Ethiopia series, highlight localized selection dynamics, possibly driven by the continued use of abacavir (ABC) in WHO-recommended pediatric regimens, notably ABC/3TC/DTG, due to its good tolerability, pediatric availability, and affordable cost. The absence of routine HLA-B*57:01 screening due to either cost constraints or low allele prevalence in the region [40], or even lack of laboratory capacity for HLA screening, may further influence ABC exposure patterns and the emergence of this mutation. Similarly, rare NRTI mutations such as F116Y or E40F typically require multiple sequential genetic events, explaining their limited distribution.

For protease inhibitors (PIs), mutations including M46I/L/V, I54V/A/S/T/L/M, I84V/A/C, and V82A/T/S/F/L/M were more frequently reported, particularly in some sub-Saharan African cohorts. These mutations confer varying degrees of resistance to boosted PIs such as lopinavir/ritonavir (LPV/r), often used in second-line pediatric therapy. Their prevalence may reflect prolonged exposure to these drugs in settings where treatment options remain available.

In contrast, mutations such as D30N, V32I, and L90M were uncommon, likely because they are selected by drugs no longer routinely used such as nelfinavir (associated with D30N) and may carry a high fitness cost [41,42,43]. Their rarity likely results from a combination of changing therapeutic practices and the epidemiological specificities of each country.

Integrase inhibitor (INSTI) resistance patterns were generally less frequent, reflecting both the relatively recent introduction of integrase inhibitors in first-line therapy and their higher genetic barrier. Mutations such as G140S/A/C/R/K were observed in several countries, with China contributing notably to the global INSTI resistance profile. E138K/A/T and R263K mutations appeared less commonly, while mutations like T66A/I/K, typically associated with elvitegravir resistance, were absent, consistent with the limited use of this molecule in many settings [44]. Several INSTI mutations were detected exclusively in China, suggesting differences in national treatment regimens or sequencing practices. Historically, integrase sequencing has not been routinely performed due to cost, technical demands, and the initially limited clinical use of integrase inhibitors [41]. As dolutegravir-based regimens continue to expand globally, routine sequencing of the integrase gene is likely to become increasingly relevant.

It should be noted that the included pediatric studies, being focused on perinatally and vertically acquired infection, did not themselves report data on adult transmission risk groups such as men who have sex with men (MSM) or people who inject drugs (PWID); the following associations between HIV-1 subtypes and predominant adult transmission modes are drawn from the broader molecular epidemiology literature rather than from data extracted in this review, and are presented here as contextual background to help interpret the regional subtype patterns observed among children [42,43]. For instance, a Portuguese molecular epidemiology study found that transmission route and subtype were independently associated, with heterosexual transmission linked to a higher probability of transmitted drug resistance and a higher representation in transmission clusters than MSM transmission, and with subtype C carrying significantly higher transmitted-resistance risk than subtype B [45]. Similarly, a phylogenetic study of the HIV-1 epidemic in Oryol Oblast, Russia, showed that subtype A (and the related sub-subtype A6) and the emerging recombinant CRF63_02A6 predominated within transmission lineages driven mainly by heterosexual and, to a lesser extent, injecting-drug-use transmission, whereas subtype B remained disproportionately linked to MSM transmission, illustrating how distinct transmission networks can shape the circulating genotype within a single country [46]. In parallel, understanding regional HIV subtype distribution is essential, as subtypes often correlate with predominant transmission modes. Heterosexual transmission is associated with CRF02_AG or CRF06_cpx in West Africa [9,16,18]. Subtype C dominates in India and parts of Brazil [23,26], while subtype B remains strongly associated with MSM transmission networks in the Americas, Europe, and South Africa [33].

These patterns underscore the importance of adapting prevention, diagnostic strategies, and treatment guidelines to regional epidemiological realities. Because pediatric HIV-1 acquisition in the included studies occured predominantly through mother-to-child transmission, associations between viral subtypes and adult-defined transmission routes should considered background epidemiological context. Althought these transmission-modes correlations may help explain the regional distribution of subtypes circulating in adult and maternal populations, they were not directly investigated in the pediatric studies included in this review. 

The present analysis provides important insight into studies investigating HIV drug resistance amongst pediatric populations. The quality assessment of retrieved data showed that traditional concerns related to measurement bias appear to have limited relevance in this context.

The drug-resistant mutations were investigated using both Sanger and next-generation sequencing technologies, which constitute the primary focus of clinical and public health decision-making.

As such, differences in sensitivity for minority variants are unlikely to significantly affect the interpretation of resistance patterns at the level considered in our study.

Similarly, while confounding factors such as treatment history, PMTCT exposure, and duration on antiretroviral therapy are biologically relevant, their impact on the descriptive assessment of resistance profiles is relatively limited.

These variables primarily influence the mechanisms underlying resistance emergence rather than the presence of major resistance mutations themselves. Therefore, when the objective is to characterize resistance broadly across pediatric populations, the influence of confounding is attenuated.

In contrast, the main methodological challenge identified in this review relates to the structure and stratification of study populations. Several studies combined ART-naïve and treatment-experienced children without clearly distinguishing between pretreatment and acquired resistance. Although this approach is consistent with the broad epidemiological reality of pediatric HIV infection, it complicates the interpretation of resistance patterns, as these two pathways are driven by distinct biological mechanisms. Pretreatment resistance is largely shaped by vertical transmission and prior maternal exposure, whereas acquired resistance reflects selective pressure under antiretroviral therapy.

This distinction is critical for the interpretation of resistance data and for informing treatment strategies. The lack of systematic stratification observed in multiple studies does not invalidate their findings but limits their analytical resolution. Future research should aim to better differentiate these resistance pathways while maintaining the representativeness of pediatric populations.

On the other hand, as a systematic narrative review, our study does not provide pooled prevalence estimates. The marked heterogeneity across included studies (in terms of design, population structure, sequencing technology, and resistance reporting formats) precluded a meaningful meta-analytic synthesis. While this limits the quantitative precision of our findings, it preserves the interpretive nuance required to capture regional variation in mutation profiles, which would be flattened by aggregation. Future studies incorporating standardized resistance reporting frameworks would facilitate more robust quantitative synthesis.

Several limitations of this review should be acknowledged. First, the search was restricted to two bibliographic databases (PubMed/MEDLINE and Scopus), and although these are major sources for the biomedical literature, relevant studies indexed exclusively in other databases or in regional/national registries may have been missed. Second, eligibility was restricted to studies published in English or French; this language restriction may have excluded relevant data published in other languages, particularly Portuguese-, Spanish-, or Russian-language studies in the literature from countries with documented pediatric HIV-1 drug resistance activity, such as Brazil and Russia, and the overall geographic representativeness of the synthesis should be interpreted with this in mind. Third, as noted above, many included studies did not clearly stratify participants into pretreatment drug resistance (PDR) and acquired drug resistance (ADR) categories; because these two pathways are governed by distinct biological mechanisms, pooling them within a single study-level estimate may obscure clinically important differences and limit the precision of cross-study comparisons. Fourth, included studies reported resistance data using heterogeneous formats, including individual mutation frequencies, aggregate class-level resistance proportions, and differing mutation panels across Sanger and next-generation sequencing platforms; this heterogeneity in reporting, rather than necessarily reflecting true biological variation, may itself exaggerate apparent differences between countries and regions and should temper direct cross-country comparisons. Fifth, and importantly, the prevalence percentages reported throughout this review are point estimates drawn from studies with markedly different sample sizes, ranging from as few as three to over a thousand participants, and confidence intervals were not consistently available for extraction; consequently, a mutation prevalence of, for example, 90% in a cohort of three children carries far greater statistical uncertainty than an equivalent prevalence in a cohort of over a thousand, even though both are presented as single percentages in the narrative synthesis. Readers should therefore interpret prevalence figures from very small studies with caution, and future primary studies and reviews in this field would benefit from routinely reporting sample sizes and confidence intervals alongside prevalence estimates to support more reliable cross-study comparison. These methodological constraints relate primarily to study design, reporting standards, and database/language coverage rather than to deficiencies in the underlying laboratory or genotyping methods, which were judged broadly comparable across studies.

Despite the importance of this issue, pediatric HIV resistance studies remain globally scarce. Ethical constraints, limited access to pediatric samples, logistical challenges, and resource limitations in high-burden countries contribute to this gap. Surveillance programs often prioritize adult populations, despite the distinct virological and therapeutic challenges of children, particularly those infected perinatally or exposed to ARVs during PMTCT interventions. This underscores the urgent need to expand pediatric HIV resistance monitoring to better guide treatment strategies and improve outcomes for this vulnerable population.

## Data Availability

All data used in this systematic review are publicly available in PubMed/MEDLINE and Scopus databases. No new datasets were generated or analyzed during the current study.

## Supplementary Materials

The following supporting information can be downloaded at https://www.mdpi.com/article/10.3390/pathogens15090958/s1: Table S1: Characteristics of the included studies; Table S2: Risk-of-bias assessment of included studies, Table S3. Prisma CheckList.

## Abbreviations

HIV	Human Immunodeficiency Virus
HIV-1	Human Immunodeficiency Virus Type 1
ARV	Antiretroviral
ART	Antiretroviral Therapy
MSM	Men who have Sex with Men
IDU	Injection Drug Use
URF	Unique Recombinant Form
CRF	Circulating Recombinant Form
WHO	World Health Organization
PRISMA	Preferred Reporting Items for Systematic Reviews and Meta-Analyses
PROSPERO	Prospective Register of Systematic Reviews
NGS	Next-Generation Sequencing
Sanger	Sanger Sequencing (method)
NRTI	Nucleoside Reverse Transcriptase Inhibitor
NNRTI	Non-Nucleoside Reverse Transcriptase Inhibitor
PI	Protease Inhibitor
INSTI/INI	Integrase Strand Transfer Inhibitor/Integrase Inhibitor
3TC	Lamivudine
d4T	Stavudine
AZT	Zidovudine
ABC	Abacavir
FTC	Emtricitabine
DTG	Dolutegravir
LPV/r	Lopinavir/Ritonavir
HLA	Human Leukocyte Antigen
PMTCT	Prevention of Mother-to-Child Transmission
ADR	Acquired Drug Resistance
PDR	Pretreatment Drug Resistance
