# Supplementary material for: HIV-1 Drug Resistance Mutations in Children: A Systematic Review of Global Genotypic Evidence (2014–2026)
Source: Pathogens. 2026 Sep 9;15(9):958. doi: 10.3390/pathogens15090958 (PMC13610180; doi:10.3390/pathogens15090958)
Supplement: Supplementary file 1 [file pathogens-15-00958-s001.zip › pathogens-4400963-supplementary.pdf]

Table S1. Characteristics of the included studies

Country	Median Age	Sample Size	Sample Type	Sequencing Methods	Classes of Antiretroviral Drugs	Subtypes	Refences
Democratic Republic of Congo	9	71	Dried blood specimens	Sanger sequencing	NRTI/NNRTI/PI/IN	-	Rubio-Garrido et al. 2021
Tanzania	-	42	Dried blood specimens	Sanger sequencing	NRTI/NNRTI	-	Shao et al.2014
Kenya	9	704	Whole blood	Next-Generation Sequencing	NRTI/NNRTI/PI	-	Abuogi and al. 2023
North America, Europe and South Africa	10	163	Plasma samples	-	NRTI/NNRTI/PI	A/A1/AG/B/C/G/F/F1/Cpx	Ross and al. 2015
Uruguay	12/15/4	3	Whole blood	-	NRTI/NNRTI/PI/IN	-	González Castro and al. 2024
Cameroon	15	246	Whole blood	Next-Generation Sequencing	NRTI/NNRTI/PI	CRF02_AG/A/F2/G/CRF13.cpx	Penda et al.2022
Brazil	12	31	-	-	IN	B/F1	Luz et al.2026
Western Kenya	<=14	480	Whole blood	Next-Generation Sequencing	NRTI/NNRTI	A1/C/D/Recombinants	Nyandiko et al. 2023
Brazil	9	38	Plasma samples	Next-Generation Sequencing	NRTI/NNRTI/PI	B/F1/C	De Azevedo and al. 2022
Ethiopia	8	554	Whole blood	Next-Generation Sequencing	NRTI/NNRTI/PI	-	Getaneh and al. 2023
Southern Tanzania	10	707	Whole blood	Next-Generation Sequencing	NNRTI/PI/IN	C/CD/CRF10_CD/D/A/AC/AD	Khamadi and al.2023

Panama	12	275	Plasma samples	Sanger sequencing	NRTI/NNRTI/PI/IN	B/G/URF_A1B	Ventosa-Cubillo and al. 2022
France	11	74	Plasma samples	-	NRTI/NNRTI/PI/IN	-	Frange and al. 2023
Kenya	10	42	Whole blood	Next-Generation Sequencing	NRTI/NNRTI	A1/A1D/A1C/D/C	Novitsky and al.2023
Equatorial Guinea	9	82	DBS	Next-Generation Sequencing	NRTI/NNRTI/PI/IN	A1/A3/B/C/D/F2/G/H/K/CRF02_AG/CRF06_cpx/CRF13_cpx/ CRF22_01A1/CRF26_AU/CRF37_cpx/CPR45_cpx	Rodríguez-Galet and al. 2022
China	12	491	Plasma samples	-	NRTI/NNRTI/PI	-	Pang and al. 2024
Zambia	13,5	273	Whole blood	Next-Generation Sequencing	NRTI/NNRTI/PI	-	Miti and al. 2020
Nigeria	6	21	plasma samples	Next-Generation Sequencing	NRTI/NNRTI	G/CRF02_AG/CRF06_cpx 5/A/AG	Ebony and al.2024
Russia	9	96	Whole blood	Next-Generation Sequencing	NRTI/NNRTI/PI	CRF63_02A6/A6/CRF02_AG/URF A6/URF B	Sivay and al. 2023
Togo	16	36	Plasma samples	-	-	-	Takassi and al. 2018
Togo	10	283	Whole blood	-	-	-	Salou and al. 2016
Uganda	2,1	317	Whole blood	-	-	A/D/C/G/CRF/URF	Kityo and al 2017

Ethiopia	9	93	DBS / DPS	Next-Generation Sequencing	-	C/ACG/AG/A	Tadesse and al. 2019
USA	11,5	451	Plasma samples	-	NRTI/NNRTI/PI	-	Van Dyke and al. 2016
South Africa	-	89	Whole blood	-	-	-	Pillay and al. 2014
Rhode Island	3	56	-	-	NRTI/NNRTI/PI	CRF02_AG/C/UN/CRF06_CPX/CRF24_BG	Rogo and al. 2015
Amazonas	3,7	117	Whole blood	Next-Generation Sequencing	-	B / C / DB / F	Andrade et al. 2017
Zimbabwe	11	702	Whole blood	Next-Generation Sequencing	-	-	Makadzange and al. 2015
sub-Saharan Africa	5,4	289	Plasma samples	Next-Generation Sequencing	-	-	Boender and al. 2016
Mali	12	33	Plasma samples	Next-Generation Sequencing	-	CRF 02_AG/CRF 06_cpx/CRF01_AE/CRF11_cpx/G/D/U	Sylla and al. 2019
India	6	36	Whole blood	-	-	C / A1	Kumar and al. 2014
Ghana	20	10	plasma samples	Next-Generation Sequencing	NRTI/NNRTI	-	Afrane and al.2025
Benin	10	47	Whole blood	Next-Generation Sequencing	NRTI/NNRTI/PI	-	Gbessin et al.2025

Table S2. Risk-of-bias assessment of included studies

Study (Country)	Design	Sample Size	Population Type	Selection Bias	Measurement Bias	Confounding	Overall Risk of Bias	Justification Summary
Rodríguez-Galet 2023 (Equatorial Guinea)	Cross-sectional surveillance	n=82 (subset of 269)	Mixed	🟡 Moderate	🟡 Moderate	🔴 High	🔴 High	Population aligned with pediatric HIVDR, but single-center and mixed populations without stratification → residual bias.
Kumar 2014 (India)	Cross-sectional	n=50 (27 analyzed)	PDR	🔴 High	🟡 Moderate	🔴 High	🔴 High	Very small, single-center cohort with limited representativeness despite relevant target population.
Nyandiko 2022 (Kenya)	Longitudinal cohort	n=480 (128 genotyped)	ADR	🟡 Moderate	🟡 Moderate	🟡 Moderate	🟡 Moderate	Failure-based genotyping aligns with ADR objective; bias now considered moderate rather than high.
Novitsky 2023 (Kenya)	Longitudinal cohort (NGS)	n=42	ADR	🟡 Moderate	🟢 Low	🟡 Moderate	🟠 Moderate–High	Target population appropriate but small highly selected cohort limits representativeness.
Abuogi 2023 (Kenya)	RCT-derived observational	n=704 (106 genotyped)	ADR	🟢 Low	🟢 Low	🟡 Moderate	🟡 Moderate	Best alignment with defined population, multicenter and structured inclusion.
Ebonyi 2024 (Nigeria)	Retrospective cohort	n≈21 (subset VF)	ADR	🔴 High	🟡 Moderate	🔴 High	🔴 High	Extremely small failure-only subset, limited generalizability.
Ventosa-Cubillo 2022 (Panama)	Cross-sectional	n=76 (67 sequenced)	ADR dominant	🟡 Moderate	🟡 Moderate	🔴 High	🔴 High	Relevant population but dominated by treated failure cases without stratification.
Rogo 2015 (USA)	Retrospective cohort	n=56 (≈40 genotyped)	Mixed	🟢 Low	🟡 Moderate	🟡 Moderate	🟡 Moderate	Full pediatric cohort, aligned with both naïve and treated populations but no stratified reporting.
Makadzange 2015 (Zimbabwe)	Cross-sectional programmatic	n=599 (subset genotyped)	ADR	🟢 Low	🟡 Moderate	🔴 High	🔴 High	Programmatic population representative, but genotyping subset and no ART stratification.
Miti 2020 (Zambia)	Cross-sectional (RCT baseline)	n=273 (77 genotyped)	ADR	🟡	🟡	🔴	🔴	Failure population consistent with ADR objective, but no

				Moderate	Moderate	High	High	stratification.
Ostankova 2023 (Vietnam)	Cross-sectional	n=61	Mixed (naïve + failure)	Moderate	Moderate	High	High	Mixed populations without analytical separation → not meta-analysable.
Van Dyke 2015 (USA)	Prospective cohort	n=234	ADR dominant	Low	Moderate	Moderate	Moderate	Strong alignment with pediatric HIVDR population but mixed exposure groups.
González Castro 2023 (Uruguay)	Case series	n=3	ADR	Critical	Moderate	Critical	Critical	No representativeness, purely descriptive.
Kityo 2017 (Uganda)	Prospective cohort	n=317	PDR + ADR separated	Low	Low	Moderate	Moderate	Well-defined pediatric population with separable PDR and ADR arms.
Salou 2016 (Togo)	Cross-sectional	n=283 (125 genotyped)	ADR	Moderate	Moderate	High	High	ART-exposed population acceptable but lacks stratification.
Agaba 2023 (Tanzania)	Cross-sectional multicenter	n=707 (74 sequenced)	ADR	Low	Low	Moderate	Moderate	Strong representativeness, resistance assessed in defined subgroup.
Pillay 2014 (South Africa)	Cross-sectional (failure cohort)	n=101 (89 genotyped)	ADR	Moderate	Moderate	High	High	Failure cohort acceptable for ADR but limited generalizability.
Ross 2015 (South Africa/Trial)	Clinical trial	n=163 (subset VF)	ADR	Moderate	Low	Moderate	Moderate	Trial population limits external validity.
Luz et al., 2026 (Brazil)	Retrospective cohort	-	Pediatric ART failure (INSTI resistance)	High	Low	Moderate	High	Very small, highly selected cohort restricted to vertically infected children with virological failure from two referral centres. Laboratory methods were robust and standardized (integrase sequencing, Stanford HIVdb, phylogenetic analysis), but the selected failure population and limited adjustment for heterogeneous treatment histories substantially reduce generalizability.

Shao 2014 (Tanzania)	Cross-sectional surveillance	n = 46 HIV-infected infants successfully genotyped	PDR, PMTCT-exposed infants	Moderate	Low	Moderate	Moderate	Population is well aligned with the study objective (PDR among HIV-infected infants identified through the regional Early Infant Diagnosis programme). However, recruitment was restricted to one zonal referral programme in northern Tanzania and the sample size was relatively small. Genotyping methodology was standardized using dried blood spots (DBS) and validated resistance interpretation. Some maternal and infant clinical factors that may influence resistance acquisition were incompletely controlled

Table S3. Prisma CheckList

Section and Topic	Item #	Checklist item	Location where item is reported
**TITLE**			
Title	1	Identify the report as a systematic review.	Page **1**
**ABSTRACT**			
Abstract	2	See the PRISMA 2020 for Abstracts checklist.	Page **1-2**
**INTRODUCTION**			
Rationale	3	Describe the rationale for the review in the context of existing knowledge.	Page **2-3**
Objectives	4	Provide an explicit statement of the objective(s) or question(s) the review addresses.	Page **3**
**METHODS**			
Eligibility criteria	5	Specify the inclusion and exclusion criteria for the review and how studies were grouped for the syntheses.	Page **3**
Information sources	6	Specify all databases, registers, websites, organisations, reference lists and other sources searched or consulted to identify studies. Specify the date when each source was last searched or consulted.	Page **3**
Search strategy	7	Present the full search strategies for all databases, registers and websites, including any filters and limits used.	Page **3**
Selection process	8	Specify the methods used to decide whether a study met the inclusion criteria of the review, including how many reviewers screened each record and each report retrieved, whether they worked independently, and if applicable, details of automation tools used in the process.	Page **4**
Data collection process	9	Specify the methods used to collect data from reports, including how many reviewers collected data from each report, whether they worked independently, any processes for obtaining or confirming data from study investigators, and if applicable, details of automation tools used in the process.	Page **3-4**
Data items	10a	List and define all outcomes for which data were sought. Specify whether all results that were compatible with each outcome domain in each study were sought (e.g. for all measures, time points, analyses), and if not, the methods used to decide which results to collect.	Page **3-4**
	10b	List and define all other variables for which data were sought (e.g. participant and intervention characteristics, funding sources). Describe any assumptions made about any missing or unclear information.	Page **3-4**
Study risk of bias assessment	11	Specify the methods used to assess risk of bias in the included studies, including details of the tool(s) used, how many reviewers assessed each study and whether they worked independently, and if applicable, details of automation tools used in the process.	Page **4-5**
Effect measures	12	Specify for each outcome the effect measure(s) (e.g. risk ratio, mean difference) used in the synthesis or presentation of results.	Not applicable: descriptive systematic review only
Synthesis methods	13a	Describe the processes used to decide which studies were eligible for each synthesis (e.g. tabulating the study intervention characteristics and comparing against the planned groups for each synthesis (item #5)).	Page **4**

Section and Topic	Item #	Checklist item	Location where item is reported
	13b	Describe any methods required to prepare the data for presentation or synthesis, such as handling of missing summary statistics, or data conversions.	Not applicable: descriptive systematic review only
	13c	Describe any methods used to tabulate or visually display results of individual studies and syntheses.	Pages 3-4
	13d	Describe any methods used to synthesize results and provide a rationale for the choice(s). If meta-analysis was performed, describe the model(s), method(s) to identify the presence and extent of statistical heterogeneity, and software package(s) used.	Pages 4–5 (narrative synthesis, no meta-analysis because of heterogeneity)
	13e	Describe any methods used to explore possible causes of heterogeneity among study results (e.g. subgroup analysis, meta-regression).	**Page 4–5** (heterogeneity explicitly discussed: study design, populations, sequencing methods, geographic contexts, reporting formats)
	13f	Describe any sensitivity analyses conducted to assess robustness of the synthesized results.	No sensitivity analyses performed
Reporting bias assessment	14	Describe any methods used to assess risk of bias due to missing results in a synthesis (arising from reporting biases).	Not applicable: no assessment of reporting bias performed
Certainty assessment	15	Describe any methods used to assess certainty (or confidence) in the body of evidence for an outcome.	Not applicable
**RESULTS**			
Study selection	16a	Describe the results of the search and selection process, from the number of records identified in the search to the number of studies included in the review, ideally using a flow diagram.	Pages **5- 6**+ Figure **1**
	16b	Cite studies that might appear to meet the inclusion criteria, but which were excluded, and explain why they were excluded.	Figure 1, Page **5-6**
Study	17	Cite each included study and present its characteristics.	Table **S1**; Pages **6-**

Section and Topic	Item #	Checklist item	Location where item is reported
characteristics			**12**
Risk of bias in studies	18	Present assessments of risk of bias for each included study.	Page **13;** Table S2
Results of individual studies	19	For all outcomes, present, for each study: (a) summary statistics for each group (where appropriate) and (b) an effect estimate and its precision (e.g. confidence/credible interval), ideally using structured tables or plots.	Page **6-13**
Results of syntheses	20a	For each synthesis, briefly summarise the characteristics and risk of bias among contributing studies.	Pages **13–17** + Table S2
	20b	Present results of all statistical syntheses conducted. If meta-analysis was done, present for each the summary estimate and its precision (e.g. confidence/credible interval) and measures of statistical heterogeneity. If comparing groups, describe the direction of the effect.	Pages **6–13**, Figures **2–6**
	20c	Present results of all investigations of possible causes of heterogeneity among study results.	Pages **4–5** and **16–17**
	20d	Present results of all sensitivity analyses conducted to assess the robustness of the synthesized results.	No sensitivity analyses were conducted.
Reporting biases	21	Present assessments of risk of bias due to missing results (arising from reporting biases) for each synthesis assessed.	Not assesed
Certainty of evidence	22	Present assessments of certainty (or confidence) in the body of evidence for each outcome assessed.	Not applicable
**DISCUSSION**			
Discussion	23a	Provide a general interpretation of the results in the context of other evidence.	Page **13–17**
	23b	Discuss any limitations of the evidence included in the review.	Page **16–17**
	23c	Discuss any limitations of the review processes used.	Page **16–17**
	23d	Discuss implications of the results for practice, policy, and future research.	Page **16–17**
**OTHER INFORMATION**			
Registration and protocol	24a	Provide registration information for the review, including register name and registration number, or state that the review was not registered.	registration number: **CRD42024512303,** Page **5**
	24b	Indicate where the review protocol can be accessed, or state that a protocol was not prepared.	Page **5**
	24c	Describe and explain any amendments to information provided at registration or in the protocol.	Not reported
Support	25	Describe sources of financial or non-financial support for the review, and the role of the funders or sponsors in the review.	Page **17**
Competing interests	26	Declare any competing interests of review authors.	Page **17**

Section and Topic	Item #	Checklist item	Location where item is reported
Availability of data, code and other materials	27	Report which of the following are publicly available and where they can be found: template data collection forms; data extracted from included studies; data used for all analyses; analytic code; any other materials used in the review.	Page **17**
